# Eosinophilic Granulomatosis With Polyangiitis: A Case Report

**DOI:** 10.7759/cureus.58211

**Published:** 2024-04-13

**Authors:** Ciji Robinson, Jasdeep S Minhas, Abraham Kisule, Hazem Zebda

**Affiliations:** 1 Internal Medicine, Henry Ford Health System, Jackson, USA; 2 Medicine, St. George's University School of Medicine, St. George's, GRD; 3 Rheumatology, Henry Ford Health System, Jackson, USA; 4 Rheumatology, Henry Ford Health System, Detroit, USA

**Keywords:** eosinophilia, eosinophilic granulomatosis with polyangiitis (egpa), egpa, churg-strauss syndrome (css), churg strauss

## Abstract

Eosinophilic granulomatosis with polyangiitis (EGPA) is a rare form of necrotizing small-to-medium vessel vasculitis that can be associated with antineutrophil cytoplasmic antibody (ANCA) positivity, asthma, and eosinophilia. We present the case of a 65-year-old male with a past medical history of asthma who presented to the emergency department with bilateral upper and lower extremity paresthesias, as well as right foot drop, persisting for a two-week duration. His lab work revealed leukocytosis of 20.6 K/uL with 12.36 K/uL of absolute eosinophils as well as elevated inflammatory markers with an erythrocyte sedimentation rate of 32 mm/hr and CRP of 7.3 mg/dL. Both c-ANCA and p-ANCA titers were also elevated at 1:320. An eventual MRI of the entire spine did not reveal any neurologic or anatomic lesions to explain the patient’s symptoms. CT imaging was also remarkable for airspace opacities involving the anterior right and bilateral lower posterior lung regions, as well as pansinusitis. A nerve biopsy showed axonopathy as well as evidence of healed vasculitis. Pulse dose steroids were started, which conferred benefits to the patient after other forms of treatment were unsuccessful. Given the rarity of EGPA, we think it is important to add new cases to the literature with a thorough discussion of the steps leading up to how the diagnosis was made.

## Introduction

Eosinophilic granulomatosis with polyangiitis (EGPA) is a rare form of necrotizing vasculitis that can be associated with antineutrophil cytoplasmic antibody (ANCA) positivity, asthma, and eosinophilia [1]. EGPA is associated with underlying asthma and upper respiratory symptoms, eosinophilia, as well as a number of other organ systems, making it a multisystem disease [2]. Annual incidence is 0.5-4.2 cases per million per year; however, this number may be underestimated as EGPA is likely underdiagnosed [3]. Given that EGPA is a multisystem disease, patients can present in many different ways; however, asthma, along with rhinosinusitis and eosinophilia, are classic findings [3]. We present the case of a 65-year-old male with a past medical history of asthma who presented to the hospital for progressively worsening upper and lower extremity paresthesias. Eventually, he was diagnosed with EGPA, contributing to the existing literature on the condition.

## Case presentation

We present the case of a 65-year-old male with a past medical history of asthma who presented to the hospital for progressively worsening upper and lower extremity paresthesias, weakness, and left foot drop over the past few weeks, causing significant impairment to his functional status. The physical exam revealed remarkable distal weakness in both lower extremities (1-2/5 strength bilaterally), diminished sensation in both lower extremities, and a right foot drop. Additionally, the patient exhibited bilateral fourth- and fifth-digit upper extremity sensory deficits and was diffusely areflexic. The skin exam yielded unremarkable findings. Lumbar puncture (LP) did not reveal evidence of Guillain-Barré syndrome (GBS), infection, or multiple sclerosis.

The patient’s lab work revealed leukocytosis of 20.6 K/uL with 12.36 K/uL of absolute eosinophils, normal neutrophil, and lymphocyte counts, as well as elevated inflammatory markers with an erythrocyte sedimentation rate of 32 mm/hr and CRP of 7.3 mg/dL (Table 1). Kidney function was within normal limits (Table 1). He received five days of intravenous immunoglobulin (IVIG) early in the hospital course as the initial presentation was concerning for GBS despite the unremarkable LP; however, this treatment did not improve his symptoms. Subsequently, the patient underwent an MRI of the entire spine, which did not reveal any neurologic or anatomic lesions to explain his symptoms. The MRI did, however, show an area suggesting possible myositis in some of the muscles surrounding the left femur. Muscle enzymes were elevated with aldolase of 10.9 U/L and creatine phosphokinase (CPK) of 819 IU/L (Table 1); however, a subsequent muscle biopsy did not show any clear evidence of myositis. The biopsy did, however, note angulated atrophic fibers, supporting the presence of an underlying neurogenic process. Of note, his muscle weakness also did not correlate with the location of possible myositis on MRI, as he had distal rather than proximal muscle weakness.

**Table 1:** Lab work ANCA, antineutrophil cytoplasmic antibody; BUN, blood urea nitrogen; CPK, creatine phosphokinase; ESR, erythrocyte sedimentation rate; GFR, glomerular filtration rate

Parameter	Value	Reference range
WBC count	20.6 K/uL	3.8–10.6 K/uL
Neutrophils (absolute)	5.97 K/uL	1.80–7.70 K/uL
Lymphocytes (absolute)	1.65 K/uL	1.10–4.00 K/uL
Eosinophils (absolute)	12.36 K/uL	0.00–0.70 K/uL
ESR	32 mm/hr	0–10 mm/hr
CRP	7.3 mg/dL	<0.5 mg/dL
BUN	12 mg/dL	10-25 mg/dL
Creatinine	0.67 mg/dL	<1.13 mg/dL
GFR	104 mL/min/1.73 m^2^	>60 mL/min/1.73 m^2^
Aldolase	10.9 U/L	1.2–7.6 U/L
CPK	819 IU/L	<250 IU/L
c-ANCA	0.263889	<1:20
p-ANCA	0.263889	<1:20

**Figure 1:**
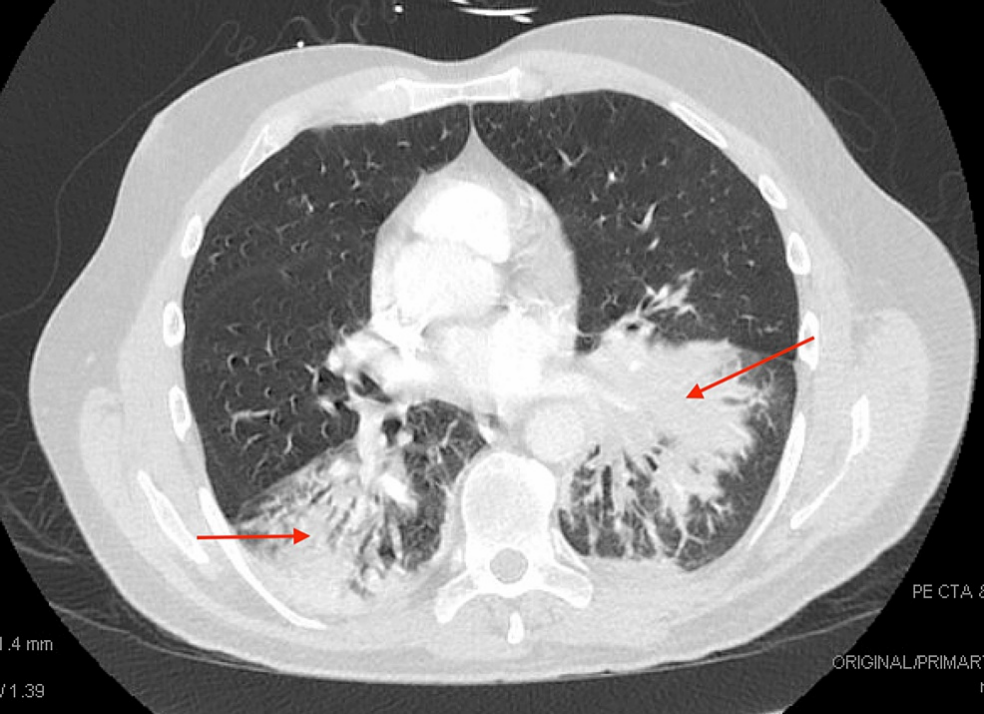
CT chest showing bilateral airspace opacities

The patient began to develop shortness of breath partway through the hospital course; thus, a CT chest was obtained, which showed airspace opacities involving the anterior right and bilateral lower lung regions (Figure 1). At this point, c-ANCA and p-ANCA titers were obtained due to concern for possible vasculitis; both were elevated at 1:320 (Table 1). Notably, a review of an MRI brain from a recent hospital admission did note chronic pansinusitis. He was then started on IV methylprednisolone 1,000 mg per day for three days with a slow taper, which provided some improvement to his weakness; extremity strength improved to 2-3/5; however, his numbness did not show significant change. Eventually, the patient underwent a radial nerve biopsy, which showed mild to moderate axonopathy as well as focal neovascularization and hemosiderin-laden macrophages associated with the surrounding small vessels, consistent with healed vasculitis. He received a rituximab infusion in the hospital and another after hospital discharge. Upon subsequent outpatient evaluation, the patient has advanced from initially being wheelchair-bound to now walking. He was started on azathioprine 50 mg daily and continues with the steroid taper.

## Discussion

Given the multisystem nature of EGPA, the patient’s chief complaint and presenting symptoms triggered the diagnostic pathway that was approached. In this patient’s case, a chief complaint of foot drop with paresthesias along with multiple neurologic findings on a physical exam led the primary thought process in the direction of a neurologic etiology. The neurologic component of EGPA has been linked to eosinophilia, causing axonal neuropathy [2]. This has been shown to be secondary to neurotoxic proteins released by the invading eosinophils that damage surrounding nerve fibers [2]. Neurologic involvement in EGPA is also often associated with ANCA positivity [4], as was the case with this patient. Peripheral nervous system involvement is also a common finding in EGPA; however, given its potential to progress to severe nerve damage, timely treatment is essential [5]. The patient’s nerve biopsy showed evidence of axonopathy, but no active eosinophils were identified given that the vasculitis was noted to be resolved.

EGPA is associated with underlying asthma, upper respiratory symptoms (sinusitis), and eosinophilia, as well as the potential for multiple other organ system involvements, including peripheral neuropathy [3]. All of the aforementioned features apply to this patient. There is no defined set of diagnostic criteria for EGPA; therefore, piecing together multiple findings while also ruling out other possibilities is how the diagnosis is achieved [4]. GBS was an initial differential diagnosis given the ascending paresthesias and areflexia; however, the LP was unremarkable, and the patient did not respond to IVIG therapy. The MRI findings also did not reveal any lesions to explain the patient’s symptoms. The patient did, however, respond to a trial of high-dose steroids initiated upon clinical suspicion of EGPA. He was diagnosed with EGPA based on a history of asthma, pansinusitis, eosinophilia, polyneuropathy, pulmonary opacities detected radiographically, ANCA positivity, and nerve biopsy findings.

## Conclusions

EGPA is often a diagnostic challenge, given its rarity and complexity. Diagnosis often requires multidisciplinary involvement since EGPA is a multisystem vasculitis, often affecting many different organ systems simultaneously. This case was additionally challenging given the fact that commonly associated organ involvements, such as skin lesions, renal dysfunction, and cardiac involvement, were not present. We believe that adding more cases like this one to the literature can help clinicians consider EGPA earlier in the diagnostic workup.
